# Accuracy of nodal staging by 18F-FDG-PET/CT in limited disease small-cell lung cancer

**DOI:** 10.1177/02184923231187279

**Published:** 2023-07-12

**Authors:** Jan Hockmann, Hubertus Hautzel, Kaid Darwiche, Wilfried Eberhard, Martin Stuschke, Clemens Aigner, Ken Herrmann, Till Plönes

**Affiliations:** 1 Department of Thoracic Surgery and Thoracic Endoscopy, 530897 University Hospital Essen-Ruhrlandklinik, Essen, Germany; 2 German Cancer Consortium (DKTK), Partner Site University Hospital Essen, Essen, Germany; 3 Department of Nuclear Medicine, West German Cancer Center, University Clinic Essen, Medical Faculty, University Duisburg- Essen, Essen, Germany; 4 Department of Pulmonary Medicine, Section of Interventional Pneumology, West German Lung Center, 530897 Ruhrlandklinik – University Hospital Essen, University Duisburg-Essen, Essen, Germany; 5 Department of Medical Oncology, West German Cancer Center, University Clinic Essen, University of Duisburg-Essen, Essen, Germany; 6 Department of Radiation Therapy, West German Cancer Center, University Hospital Essen, Essen, Germany

**Keywords:** SCLC, FDG-PET/CT, nodal staging, EBUS, mediastinal staging, diagnostic accuracy

## Abstract

**Background:**

Small-cell lung cancer (SCLC) is highly aggressive with a nearly incurable disease in most cases. The most important prognostic factor is the status of the mediastinal lymph nodes. Only a small proportion of patients can be diagnosed at early stages and directed to curative multimodal treatment. Therefore, accuracy of nodal staging by (18F)-Fluoro-2-deoxyglucose (FDG) positron emission tomography (PET) computed tomography (18F-FDG-PET/CT) in (very) limited disease SCLC, although not well investigated, is highly important.

**Methods:**

Treatment naive, non-bulky patients treated or diagnosed with SCLC between June 2012 and April 2020 with complete data including FDG-PET/CT and invasive mediastinal staging were retrospectively analyzed (*n* = 19). Sensitivity, specificity, negative predictive value (NPV), positive predictive value (PPV) and accuracy of mediastinal lymph node staging of 18F-FDG-PET/CT was calculated.

**Results:**

The FDG-PET/CT showed a sensitivity of 91%, and the specificity was calculated as 87.5%. In this cohort, the disease prevalence in lymph nodes was 58% (*n* = 11). Positive predictive value was 91%, NPV 88% and accuracy calculated at 89%. One patient was upstaged from single-level N2 to multilevel N2. In one patient, upstaging in invasive staging was performed from N2 to N3, and one patient was downstaged from N1 to N0.

**Conclusions:**

FDG-PET/CT is a valuable tool for the detection of distant metastases, but in mediastinal staging of SCLC some limitations might remain. Invasive methods remain the gold standard. Therefore, the mediastinal lymph nodal status of patients with SCLC screened for multimodal treatment should be further evaluated by additional invasive techniques to verify the exact N-staging and to optimize treatment stratification.

## Introduction

Lung cancer (LC) is one of the most common malignancies in the Western world, and the cancer-specific survival of LC is still low.^1^ The most prevalent histological subtype of LC is non-small cell lung cancer (NSCLC) which accounts for up to 85% of LCs. The more aggressive subtype of small-cell lung cancer (SCLC) accounts for up to 15% of LC cases.^2^ Improvement of diagnostics and therapies has been achieved over time mostly in NSCLC,^3^ but the last three decades have shown only little progress in treatment of SCLC.^4^ Unfortunately, most patients with SCLC were diagnosed with a local advanced or even metastatic stage (extensive disease). Since the last decades, the established standard treatment for patients with extensive disease is platin-based chemotherapy (since the IMpower133 trial in combination with PD-L1 targeted immune checkpoint inhibitor Atezolizumab), but median overall survival is still limited by early recurrence.

Staging is essential for the outcome in all stages of SCLC,^5^ because prognosis correlates not only with the existence of distant metastases but also with nodal stage.^5,6^ Especially the small subgroup of patients with very limited disease (VLD) may draw profit from multimodal therapy in a curative intend with outcomes up to 25% long-term survival.^4^ An adequate mediastinal staging is the key to multimodal treatment of SCLC by identifying patients benefitting from local therapy.^7^ The (18F)-Fluoro-2-deoxyglucose (FDG) positron emission tomography (PET) computed tomography (CT) (FDG-PET/CT) is highly sensitive in the detection of distant metastases but the exact value for mediastinal staging in SCLC is unknown.^8,9^ Comparative studies have been performed for NSCLC,^10^ but studies for SCLC are lacking.^11^ Therefore, the aim of the study is to compare the image-based nodal staging by FDG-PET/CT with the results of invasive staging procedures by endobronchial ultrasound-guided transbronchial needle aspiration (EBUS-TBNA) and/or mediastinoscopy (MSC) to evaluate the role of FDG-PET/CT in mediastinal nodal staging of SCLC.

## Methods

### Patients

**Figure 1.**
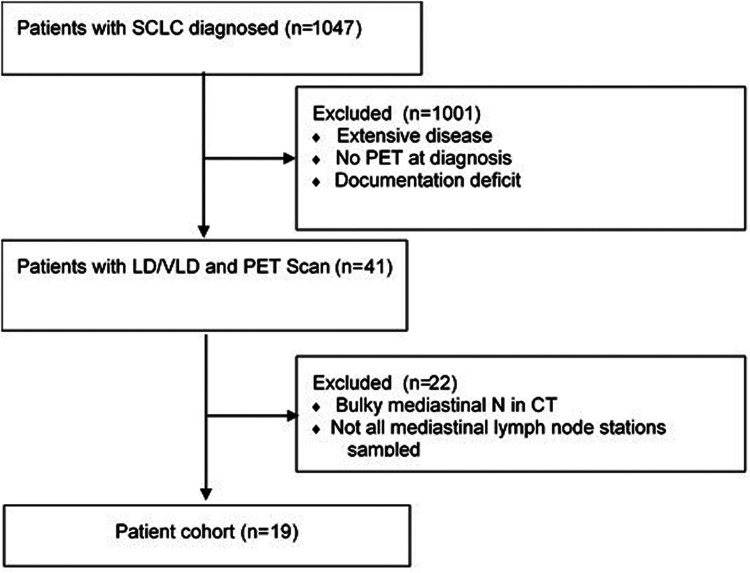
Patients cohort (selection of patients included and analyzed). SCLC: small cell lung cancer; PET: positive emission tomography; LD: limited disease; VLD: very limited disease; N: lymph nodes; CT: computer tomography.

All consecutive patients treated and/or diagnosed with SCLC in the Department of Thoracic Surgery and Thoracic Endoscopy of the Clinic between June 2012 and April 2020 were included. Patients with bulky *tumor* disease in initial CT and without pre-therapeutic performed FDG-PET/CT were excluded from further analysis to avoid bias. We also excluded patients diagnostic EBUS-TBNA only without systematic lymph node sampling (Figure 1). Therapy-naive patients with histopathological confirmed SCLC, without distant metastasis and without bulky disease were included. Data concerning medical history, histological results from EBUS and/or MSC and results from FDG-PET/CT scan were extracted from the medical database of the center. Patients with mixed tumor (SCLC/NSCLC) were excluded. Tumors located within ≤2 cm the tracheobronchial tree or adjoined to the pericardium, mediastinum, or the spine were defined as centrally located.^12^

### Staging

Staging was based upon the 8th TNM Edition of the Union Internationale contre le Cancer (UICC). Patient included prior to 2017 were restaged according to the 8th TNM edition. The various lymph node stations were grouped according to the classification of Mountain and Dressler.^13^

### FDG-PET/CT

The procedure was performed before the EBUS-TBNA or any other invasive procedure took place. Patients were fasting at least 6 hours before (18F)-Fluoro-2-deoxyglucose administration. Blood glucose was controlled and below 160 mg/dl before injection of FDG; 66 ± 11 min after injection of 288 ± 46 MBq a diagnostic PET/CT scan was acquired (Biograph mCT 128, Siemens Healthcare, Erlangen, Germany; Biograph Duo, Siemens Healthcare, Erlangen, Germany).

All PET/CT images were interpreted by an experienced expert in nuclear medicine (H.H., certified board member with 21 years of experience in PET/CT reading) who was blinded to the final clinical N stage as derived by either EBUS-TBNA or MSC.

### Endobronchial ultrasound bronchoscopy

Endobronchial ultrasound bronchoscopy was performed under general anesthesia. In case of a contraindication to general anesthesia, sedation of the patient or only local anesthetics was used. Imaging of the mediastinum was achieved with an Olympus system (Olympus Ltd, Tokyo, Japan). Combined bronchoscopy and transesophageal ultrasound was commonly used for identifying enlarged lymph nodes as well as unsuspicious lymph nodes. For fine needle aspiration guided puncture of lymph node stations a 22-gauge needle was routinely used. Evaluation of Station 2R/L, 4R/L, 7 and 10 was routinely performed as a minimum. Diagnosis was verified by immunohistochemistry.

### Mediastinoscopy

The procedure was performed under general anesthesia. Through a small jugular incision, a video-mediastinoscope was used to evaluate the mediastinum for Station 2R/L, 4R/L, and 7 as a minimum. Magnification by using video techniques enhanced this procedure. Small samples or whole lymph nodes were excised. Probes were evaluated by an experienced pneumo-pathologist and were verified by immunohistochemistry.

### Definition of true positive and negative

True positive was defined as same N-stage in the evaluating invasive method compared to FDG-PET/CT and N-stage larger than zero. True negative was defined as N-stage zero in both clinical evaluating methods. False negative was defined as a lower N-stage in FDG-PET/CT than in invasive methods. False positive outlined higher N-stage in FDG-PET/CT than in EBUS or MSC.

### Statistical analysis

MedCalc statistical software, Version 19.2.1 (MedCalc software, Broekstraat 52, 9030 Mariakerke, Belgium) was used for all statistical analyses. The median is given with range, and the average is given with standard deviation. Sensitivity was calculated by: $Sensitivity=\frac{\mathrm{True}\;\mathrm{positives}}{\mathrm{True}\;\mathrm{positives}+\mathrm{False}\;\mathrm{negatives}}$ . Specifity was calculated by: $Specifity=\frac{\mathrm{True}\;\mathrm{negatives}\;}{\mathrm{True}\;\mathrm{True}\;\mathrm{negatives}\;+\;\mathrm{False}\;\mathrm{positives}\;}$ . Positive predictive value (PPV) was calculated by $PPV=\frac{\mathrm{Sensitivity}\;*\;\mathrm{prevalence}\;}{\mathrm{Sensitivity}*\mathrm{prevalence}\;+\;(1-\mathrm{specificity})\;*\;(1-\mathrm{prevalence})}$ . Negative predictive value (NPV) was calculated by $NPV=\frac{\mathrm{Specificity}\;*\;(1-\;\mathrm{prevalence})}{\mathrm{Specificity}\;*\;(1-\mathrm{prevalence})+\;(1-\mathrm{sensitivity})\;*\;\mathrm{prevalence}\;}$ . Accuracy was calculated by *Accuracy* = *Sensitivity* * *Prevalence* + *Specificity* * (1 − *Prevalence*). Confidence intervals are given for sensitivity, specificity, accuracy, PPV, and NPV.

## Results

We screened 1047 patients with SCLC diagnosed and/or treated in our institution. A total of 217 patients had an FDG-PET/CT and a complete invasive staging before starting any therapy. We excluded 198 patients with bulky disease and analyzed the remaining 19 patients. The characteristics of the patient collective are shown in Table 1. We had a reported active smoking history in most patients (*n* = 13).

**Table 1.** Clinical characteristics of patients.

Characteristic	
Median age (years), mean ± SD	67 (range 49–79)
Sex (male), *n* (%)	10 (52.6)
Median FEV1	78.1 (range 50%–100%)
Median LDH	216.5 (range 135–392)
Median SUV_max_ mediastinal lymph node	4.7 (range 0–12)
Median SUV_max_ primary tumor	6.8 (range 3.2–18.3)
Side of primary tumor (right), *n* (%)	12 (63.2)
Location of primary tumor (peripheral), *n* (%)	16 (84.2)

CO*Hb*: carboxyhemoglobin; FEV1: forced expiratory volume in one second in percent of the index value; LDH: lactate dehydrogenase U/L; SUV_max_: maximal standardized uptake value.

Only three patients (15%) had a centrally located tumor, whereas the remaining had a peripheral lesion. Primary tumor staging revealed cT1a/b/c in nine patients (47%), cT2a in six patients (31%), cT3 (15%) in three patients, and cT4 (7%) in one patient in FDG-PET/CT.

The mediastinal lymph node staging based on the preliminary initial CT scan revealed cN0 in 12 patients (63%), whereas three patients had ipsilateral cN1 situation (15%). There were also three patients with a cN2 (15%) and one patient (7%) with a contralateral hilar mediastinal lymphadenopathy indicating cN3 status (Table 2). Median time between the initial CT scan, and the FDG-PET/CT was 14 days (range 0–102 days).

**Table 2.** Up-/downstaging of mediastinal lymph nodes in the patient’s cohort.

Patient	cN^(CT scan)^	cN^(18F−FDG PET CT)^	cN^(invasive Biospy)^	Up-/downstaging*	Location of tumor
1	2	2	2	(Upstaging*)	Right upper lobe
2	0	3	3		Left central
3	1	2	2		Left upper lobe
4	0	2	2		Right upper lobe
5	1	2	2		Middle lobe
6	2	0	0		Right central
7	0	1	1		Left upper lobe
8	3	2	2		Left upper lobe
9	2	0	0		Right central
10	1	3	3		Left upper lobe
11	0	2	3	Upstaging	Right upper lobe
12	0	1	1		Right lower lobe
13	0	1	0	Downstaging	Right upper lobe
14	0	0	0		Left upper lobe
15	0	0	0		Right upper lobe
16	0	0	0		Left lower lobe
17	0	1	1		Right lower lobe
18	0	0	0		Right upper lobe
19	0	0	0		Middle lobe

PET: positron emission tomography.

*Upstaging from single level N2 to multilevel N2.

FGD-PET CT improves staging compared to conventional CT and enables better clinical decision-making.

**Figure 2.**
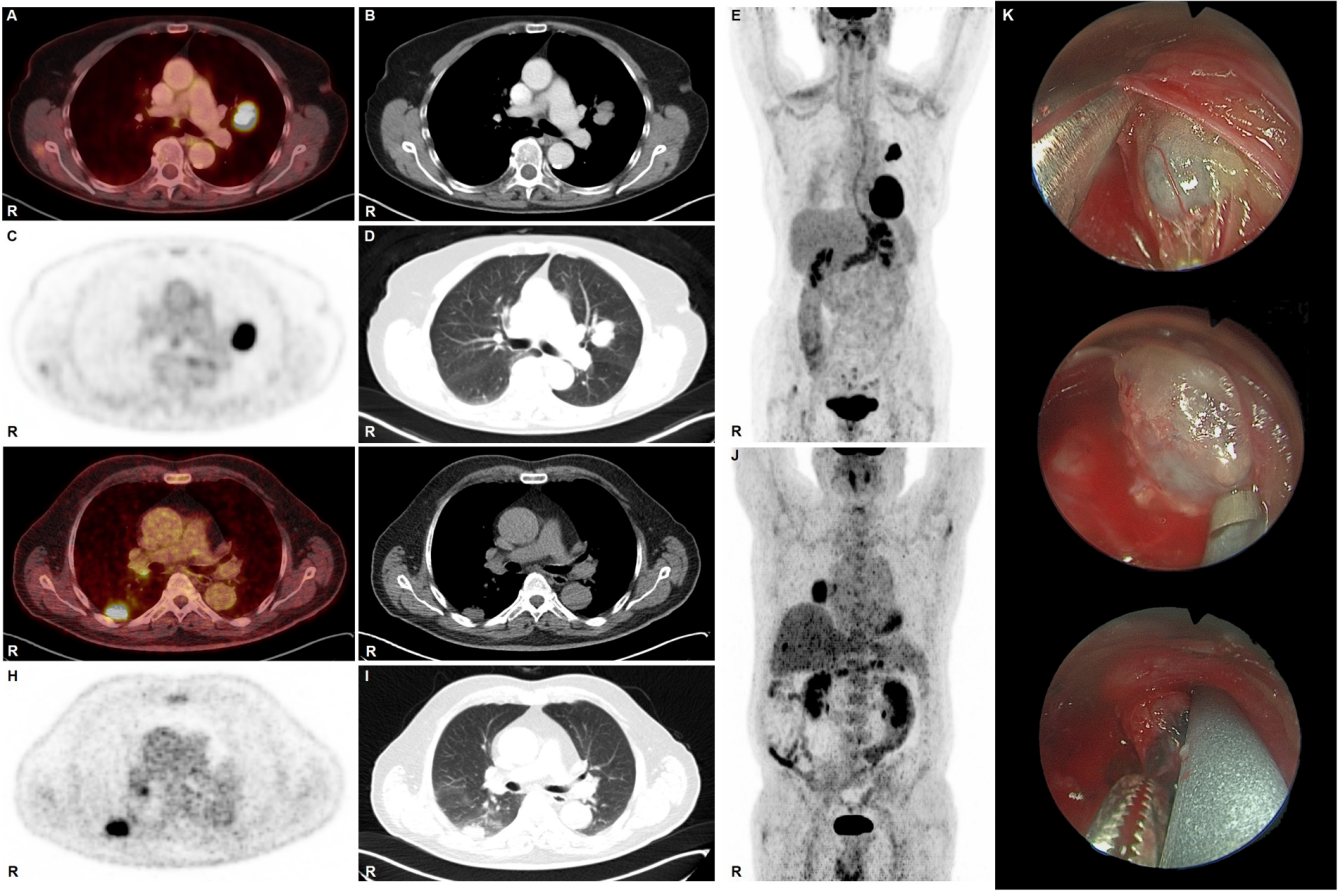
(a–e) Patient with nodal stage cN0 (true negative) in FDG-PET/CT: female, age 77 years, SCLC, cT1c pN0 (0/6) cM0, and very limited disease. SUVmax primary 18.3, SUVmax true negative lymph nodes <2.5. (A) FDG PET/CT fusion, (B) CT chest soft tissue window, (C) PET attenuation-corrected emission, (D) CT chest lung window, (E) PET maximum intensity projection (MIP). Patient received EBUS-TBNA and subsequently lobectomy of the upper left lobe plus ipsilateral lymphadenectomy in curative intent. The patient died 5 months later. (f–j) Patient with nodal stage cN1 (true positive) in FDG-PET/CT: male, age 74 years, SCLC pT1b cN1 (1/3) M0, and limited disease. SUVmax primary 10.4, SUVmax true positive lymph node 4.2. (f) FDG PET/CT fusion, (g) CT chest soft tissue window, (h) PET attenuation-corrected emission, (i) CT chest lung window, (j) PET maximum intensity projection (MIP). Patient underwent EBUS-TBNA which proved stage cN1. Subsequently, multimodal treatment was initiated including induction chemotherapy and radiochemotherapy followed by lobectomy of the right lower lobe plus ipsilateral lymphadenectomy in curative intent. Histopathology of the resected specimen revealed ypT0 pN0(0/14) cM0. At the last follow-up, 57 months after initial diagnosis the patient remained in complete remission. (k) Intraoperative pictures of lymph nodes during video-assisted mediastinoscopic lymphadenectomy (VAMLA). SCLC: small cell lung cancer; EBUS-TBNA: endobronchial ultrasound-guided transbronchial needle aspiration.

The mediastinal lymph node staging based on FDG-PET/CT was revealed only in seven patients cN0 (37%) (Figure 2A–E). Four patients were diagnosed with cN1 (21%) (Figure 2F–J), six patients had cN2 (32%) and two patients had cN3 (11%). One patient with a cN0 in the previous CT scan was upstaged to cN1 by FDG-PET/CT, and one patient was upstaged from cN0 to cN2. Only one patient with cN1 in the previous CT scan was upstaged to cN2 by FDG-PET/CT, and one patient with cN2 was upstaged to cN3.

A comparison of the FDG-PET/CT staging with the biopsy of the mediastinal lymph nodes revealed that all patients with FDG-PET/CT cN0 were correctly diagnosed. Regarding the four patients with cN1 in FDG-PET CT histological biopsy revealed one downstaging to cN0 and no upstaging. In contrast, there was one patient with cN2 in FDG-PET CT who was upstaged to cN3 in EBUS-TBNA. Another patient with single level N2 in FDG-PET CT showed to be a multilevel N2 in EBUS-TBNA. The two patients with cN3 in FDG-PET CT were correctly staged as compared to the histological reference.

Based on the gold standard of invasive mediastinal staging the following parameters were calculated.

The FDG-PET showed a sensitivity of 91%, and the specificity was calculated as 87.5%. In this cohort, the disease prevalence in lymph nodes was 52%. Positive predictive value was 81%, NPV 87% and accuracy was calculated to 84%.

The CT scan alone showed a sensitivity of 54%, and the specificity was calculated as 100%. In this cohort, the disease prevalence in lymph nodes was 52%. PPV was 100%, NPV 61% and accuracy calculated to 73%.

## Discussion

Due to its aggressive behavior and the tendency for early metastatic spread, SCLC has still a poor prognosis. At the time of diagnosis, most patients are already in an advanced stage of disease and systemic chemotherapy is the only option. In contrast, patients with LD or even VLD have the option of radiochemotherapy or surgery in a multimodal setting. For these patients who reported median overall survival is much higher, and there is a chance for cure. Unfortunately, this only applies to a minority of patients with SCLC. However, the increasing introduction of LC screening may also lead to an increase in the diagnosis of SCLC in the early stages. In these patients, accurate mediastinal staging is fundamentally important for therapy selection. The mediastinal lymph nodes are the most important predictor for survival and false positive or false negative findings can jeopardize optimal therapy planning.

We demonstrated in our study the evidence of up- and downstaging of the nodal status of patients LD/VDL in SCLC by using non-invasive FDG-PET/CT. Thus, the addition of invasive staging in SCLC is very necessary for treatment planning.

Especially for positive nodal results in FDG-PET/CT further verification still seems to be needed. False positive status of mediastinal lymph nodes may be caused by inflammatory processes like post-stenotic pneumonia, which is common in central tumors. The specificity may be too low to indicate usage of FDG-PET/CT alone as the staging method of SCLC compared to studies evaluating EBUS-TBNA indicating a higher specificity.^14,15^ A recent study evaluated the benefits of increased incorporation of EBUS and PET/CT and indicated a more precise stratification.^16^ The data suggest that FDG-PET/CT alone could not match up to that gain of stratification. Compared to CT alone an improvement in accurate staging by incorporating PET/CT scans was shown.^17,18^ Invasive mediastinal staging methods like EBUS-TBNA or MSC still remain the gold standard in the precise evaluation of nodal involvement. Due to the retrospective study design, small patient number and evaluation of existing reports, the study may be limited.

## Conclusions

FDG-PET/CT is a valuable tool for the detection of distant metastases, but in mediastinal staging of SCLC some limitations might remain. EBUS-TBNA and MSC remain the gold standard, as we were able to show more different results of the invasive staging techniques compared to FDG-PET/CT. Therefore, the mediastinal lymph nodal status of patients with SCLC screened for multimodal treatment should be further evaluated by additional invasive techniques to verify the exact N-staging and to optimize treatment stratification.
